# Addressing psychosocial needs in patients with Long-COVID (PsyLoCo-Study): study protocol of a pilot-study of a specialized modular intervention

**DOI:** 10.3389/fpsyt.2024.1305691

**Published:** 2024-03-06

**Authors:** Christine Allwang, Tamara Frank, Paul Bruckmann, Andreas Dinkel, Marius Binneboese, Hannah Wallis, Melanie Elgner, Katrin E. Giel, Marisa Schurr, Harald Gündel, Lisa Wedekind, Julia Kuhn, Claas Lahmann, Anne-Maria Müller, Pauline Beckmann, Janka Massag, Rafael Mikolajczyk, Florian Junne

**Affiliations:** ^1^ Department of Psychosomatic Medicine and Psychotherapy, Klinikum rechts der Isar, Technical University Munich, Munich, Germany; ^2^ Department of Psychosomatic Medicine and Psychotherapy, Otto-von-Guericke-University Magdeburg, Magdeburg, Germany; ^3^ Department of Psychosomatic Medicine and Psychotherapy, Eberhard Karls University Tuebingen, Tuebingen, Germany; ^4^ Department of Psychosomatic Medicine and Psychotherapy, Ulm University, Ulm, Germany; ^5^ Department of Psychosomatic Medicine and Psychotherapy, Albert-Ludwigs-University Freiburg, Freiburg, Germany; ^6^ Medical Faculty, Institute of Medical Epidemiology, Biometrics and Informatics, Martin-Luther-University Halle-Wittenberg, Halle (Saale), Germany; ^7^ German Center for Mental Health (DZPG), partner site Halle-Jena-Magdeburg, Halle, Germany; ^8^ German Center for Mental Health (DZPG), partner Site Halle-Jena-Magdeburg, Magdeburg, Germany

**Keywords:** COVID-19, Long-COVID, post-acute COVID-19 sequelae, randomized controlled trial, protocol, psychosocial needs, psychotherapy, pilot study

## Abstract

**Introduction:**

After an acute infection with the corona virus 10-20% of those affected suffer from ongoing or new symptoms. A causal therapy for the phenomenon known as Long/Post-COVID is still lacking and specific therapies addressing psychosocial needs of these patients are imperatively needed. The aim of the PsyLoCo-study is developing and piloting a psychotherapeutic manual, which addresses Long/Post-COVID-related psychosocial needs and supports in coping with persistent bodily symptoms as well as depressive or anxiety symptoms.

**Methods and analysis:**

This pilot trial implements a multi-centre, 2-arm (N=120; allocation ratio: 1:1), parallel group, randomised controlled design. The pilot trial is designed to test the feasibility and estimate the effect of 1) a 12-session psychotherapeutic intervention compared to 2) a wait-list control condition on psychosocial needs as well as bodily and affective symptoms in patients suffering from Long/Post-COVID. The intervention uses an integrative, manualized, psychotherapeutic approach. The primary study outcome is health-related quality of life. Outcome variables will be assessed at three timepoints, pre-intervention (t1), post-intervention (t2) and three months after completed intervention (t3). To determine the primary outcome, changes from t1 to t2 are examined. The analysis will be used for the planning of the RCT to test the efficacy of the developed intervention.

**Discussion:**

The pilot study will evaluate a 12-session treatment manual for Long/Post-COVID sufferers and the therapy components it contains. The analysis will provide insights into the extent to which psychotherapeutic treatment approaches improve the symptoms of Long/Post-COVID sufferers. The treatment manual is designed to be carried out by psychotherapists as well as people with basic training in psychotherapeutic techniques. This approach was chosen to enable a larger number of practitioners to provide therapeutic support for Long/Post-COVID patients. After completion of the pilot study, it is planned to follow up with a randomized controlled study and to develop a treatment guideline for general practitioners and interested specialists.

**Trial registration:**

The pilot trial has been registered with the German Clinical Trials Register (Deutsches Register Klinischer Studien; Trial-ID: DRKS00030866; URL: https://drks.de/search/de/trial/DRKS00030866) on March 7, 2023.

## Introduction

### Background and rationale

Since the first outbreak of SARS-CoV-2 in December 2019 over 766 million cases of infections with the corona virus have been officially confirmed worldwide (1). Many of those who have overcome the acute COVID-19 infection report continuing symptoms extending several weeks or months. This condition, which is characterized by new or ongoing symptoms four weeks or more after the start of acute COVID-19, is commonly referred to as Post-COVID-19 or Long-COVID (2). In the recent literature, the resulting pathological condition has also been referred to as “long COVID/post-acute COVID” and “chronic post-COVID syndrome” (3). According to the WHO an estimated 10-20% of those who had been infected with SARS-CoV-2 are suffering from Long/Post-COVID (4). However, the estimates span between 2.3% and 72.5% (5, 6).

Like other post-acute infection syndromes, Long/Post-COVID condition is highly complex (7). Studies report a large variety of up to over 200 symptoms (8–10). Among the most prevalent symptoms are fatigue, headaches, attention disorder, dyspnea and hair loss (9, 11).

Mental health problems are also prominent, as up to 78% of survivors develop neuropsychiatric Long/Post-COVID symptoms, most commonly symptoms of depression, anxiety, and cognitive deficits (12–14).

Current theories regarding the pathogenesis of Long/Post-COVID hypothesise processes of immune dysregulation, microbiota dysbiosis, immunological processes, blood clotting and endothelial abnormalities or dysfunctional neurological signalling (15). Sufficient scientific evidence to adequately confirm postulated pathogenetic mechanisms is still lacking.

Apart from clinical variables, several psychological factors like psychological distress, loneliness, worry, expectations associated with COVID-19 (16) or resilience (17) are significant predictors of Long/Post-COVID symptom burden. Several recent studies focus on the psychosocial perspective and emphasize it as important as potential biological factors (18, 19).

Patients’ symptom trajectory usually shows an undulated pattern. While for many symptoms decreasing prevalences are recorded over time, stable or increasing symptom prevalences have also been observed (10). Long/Post-COVID is often associated with reduced quality of life and an inability to return to work (20–22). Furthermore, the disease impact on patients’ lives often increases 6 months after the infection (10).

In addition to the impairment of the individual’s health and quality of life, these symptoms pose a general and considerable socioeconomic burden (20). In the United States alone, Long/Post-COVID has been estimated to lead to an annual loss of income of approximately 50 billion $US. The annual cost of treatment per person has been estimated to be 9000 $US (23).

Given the association between psychological variables with Long/Post-COVID symptom burden as well as the enormous impact Long/Post-COVID can have on individuals, psychological and psychosocial approaches should be an incremental component of treatment. Although isolated nonspecific psychological interventions are a part of several intervention studies (24), to our knowledge there is no existing psychotherapeutic intervention tailored specifically to the psychosocial needs of Long/Post-COVID patients.

Therefore, we developed a psychotherapeutic manual addressing psychosocial needs as well as bodily symptoms of Long/Post-COVID patients. Explicit focus is put on coping strategies, bodily-distress management, management of affective symptoms and problems regarding patients’ social life and work life. The development of the intervention is based on expert knowledge and existing manuals for short-term psychotherapeutic interventions (25–27). Besides results from data analyses of two German COVID-19-cohorts, interviews with Long/Post-COVID-patients regarding their psychosocial needs and preferences have also been incorporated in the development of this intervention.

## Objectives

The aim of the study is to develop and pilot test a newly developed manualized psychotherapeutic intervention, which addresses in particular psychosocial needs and preferences of patients suffering from Long/Post-COVID.

## Trial design

The pilot trial is conducted as a multi-centre, 2-arm (allocation ratio: 1:1), parallel group, randomised and controlled study.

## Methods: participants, interventions and outcomes

The article was written in adherence to the SPIRIT reporting guidelines (28).

A council of Long/Post-COVID patients was formed and regular meetings were held to discuss the methods especially the content of the therapeutic intervention.

### Study setting

The study is conducted at six locations across Germany, which constitute the PsyLoCo-consortium. Participating institutions are five psychosomatic university clinics in Munich (Technical University; TU), Magdeburg, Tuebingen, Ulm and Freiburg, as well as the university Halle (Saale). The latter is responsible for the overall data management and statistical analysis. Therapeutic sessions are held in all of the psychosomatic sites.

### Eligibility criteria

Eligibility criteria for participants are as follows:

*Inclusion criteria:* 1) Age ≥ 18y. 2) Previous SARS-CoV-2 infection confirmed either by a PCR-positive test result or positive self-rapid test. 3) At least one present Long/Post-COVID-symptom (persisting min. 4 weeks after initial infection). 4) Ability to provide informed consent. 5) Availability during intervention period. 6) Suitable place of residence to attend the appointments at one of the study locations.

*Exclusion criteria:* 1) Severe psychiatric disorder according to ICD-10. 2) Current suicidality. 3) Current psychotherapeutic treatment or having received psychotherapeutic treatment for Long/Post-COVID. 4) Insufficient understanding of spoken or written German.

All therapists will be psychologists or physicians with appropriate psychotherapeutic training and they will receive training in conducting the interventions according to the manual.

### Who will take informed consent?

Study participants will be informed in detail about the study. Potential effects and benefits as well as possible side effects are explained. Additionally, participants are informed about data security and their participant rights. Study personnel will then obtain written informed consent before pre-intervention assessments (t1).

### Additional consent provisions for collection and use of participant data and biological specimens

A study module investigating intraindividual changes in blood cytokines between t1 and t2 is optional. Separate informed consent will be obtained from patients who agree to participate.

## Interventions

### Explanation for the choice of comparators

Due to the randomized assignment to either the intervention group or the control group, any systematic differences and changes regarding symptom burden should be attributed to group affiliation and having received the psychotherapeutic treatment or not. As controls, a waiting list group was chosen as not offering the intervention to controls at a later time point would likely result in poor participation.

During the trial, both study groups are allowed to use regular medical care for Long/Post-COVID-symptoms, i.e. general practitioners or medical specialists (e.g. neurologists, nephrologists, cardiologists or physiotherapists). Participants who initiate psychotherapeutic treatment other than within the study intervention will be excluded.

### Intervention description

The psychotherapeutic intervention is based on insights from data analysis of two patient cohorts and a set of patient interviews focusing on psychosocial needs and preferences of patients suffering from Long/Post-COVID conditions. The therapeutic manual was developed in an iterative process by a team of interdisciplinary experts (i.e. clinical psychologists, psychotherapists, residents, medical specialists). Additional feedback and comments of a group of patient representatives was also implemented in the development of the intervention. The intervention will be carried out as individual therapy in 12 sessions with one session per week. The first therapeutic sessions were held mid-March 2023. The last follow-up-measures are planned end of November 2023. The 12-session intervention is divided into four modules. Module 1 focuses on coping and distress management, module 2 on somatic symptoms and pain, module 3 on chronic fatigue and affective symptoms and module 4 on stresses regarding social and work life. The psychotherapeutic manual contains treatment techniques from cognitive-behavioral, psychodynamic and systemic psychotherapeutic approaches and techniques (i.e. psychoeducation, work sheets, resource work; **Table 1**). The implementation of the manual offers a certain degree of flexibility. In addition to the components that are always carried out for each participant, there are optional components that can be applied according to individual needs.

**Table 1:** Session focus and interventions for each therapeutic session.

Session	Session focus	Interventions
1	•Therapeutic relationship •Psychoeducation Long/Post-COVID	•Documentation symptoms •Documentation course of disease •Psychoeducation Long/Post-COVID
2	•Therapeutic relationship •Defining goals of therapy •Introduction relaxation techniques	•Introduction PMR •Introduction MBSR
3	•Resource work	•Resource list/case
4	•Mind-body interplay •Psychosomatic disease model	•Psychoeducation (e.g. lemon exercise) •Individual barrel model
5	•Interplay attention and bodily symptoms	•Psychoeducation •Strategies for attention re-focus
6	•Mindfulness and bodily symptoms	•Introduction mindful perception
7	•Long/Post-COVID and depressive symptoms	•Emotional triangle •Cognitive defusion
8	•Long/Post-COVID and personal values	•Activity planning according to personal values
9	•Long/Post-COVID and fatigue	•Strategies for handling of fatigue symptoms
10	•Long/Post-COVID at work	•Work anamnesis •Personal resources at work
11	•Performance expectations in the context of work	•Strategies for adjustment of expectations in the context of work
12	•Relapse prevention and feedback	•Summary of therapeutic insights •Identifying early warning signs •Identifying high risk situations

### Criteria for discontinuing or modifying allocated interventions

A discontinuation of treatment or modification of the intervention will be initiated if a patient suffers acute or severe symptom deterioration including suicidality. In this case appropriate treatment will be initiated. The intervention can also be terminated by the participants themselves at any point in time.

### Strategies to improve adherence to interventions

Within the participant information and informed consent form, patients are made aware of the importance of regular and active participation in the therapy sessions. Patients in the waitlist control group will be called after six weeks of waiting to ensure continued participation.

Via standardized checklists, which specify the contents of each therapy session, adherence to the manual is measured. The therapists indicate which parts of the manual were carried out and which were not. If parts could not be carried out, the reasons for this are recorded, e.g. the content of the lesson was too extensive for the participant due to concentration problems.

### Relevant concomitant care permitted or prohibited during the trial

Any kind of medical care and therapy is allowed during the trial except psychotherapy (beside the study intervention).

### Provisions for post-trial care

It is hypothesized that participants will benefit from the intervention and that Long/Post-COVID-symptoms will improve. Harm from trial participation is not expected. If applicable, participants will be supported in finding appropriate follow-up treatment such as inpatient psychosomatic treatment or outpatient psychotherapy.

### Outcomes

The primary outcome of the study is the feasibility of the developed intervention. We assess whether it is possible to recruit patients, to conducte the developed intervention with them, and measure the outcomes.

In addition, an estimate of the effect size in preparation of an RCT will be conducted, and the feasibility of the corresponding assessments investigated. For the later RCT the primary outcome is health-related quality of life (measured via Short-Form Health Survey-12; SF-12). Secondary outcomes are somatic symptoms (Patient Health Questionnaire-15; PHQ-15 and Long/Post-COVID symptoms), depressive symptoms (PHQ-9), anxiety (Generalized Anxiety Disorder-7; GAD-7), fatigue (Fatigue Assessment Scale; FAS), perceived stress (Perceived Stress Questionnaire-20; PSQ-20), work ability (Work Ability Score; WAS), mental well-being (WHO-Five Well-Being Index; WHO-5), illness perception (Revised Illness Perception Questionnaire; IPQ-R), symptom-related thoughts, feelings and behaviour (Somatic Symptom Disorder - B Criteria Scale; SSD-12) and work stress (Psychosocial Safety Climate-4; PSC-4). The questionnaires are used in both groups at baseline (t1), directly post-intervention (t2) and six months after baseline assessment (t3). The analysis of intervention effects compared to control group is only conducted at the end of the intervention in the primary intervention group (comparison of intra-individual improvement). Later measurements are only used to assess if the effects in the early and late (after waiting) intervention group are similar or whether the effects are maintained after the intervention phase. Additionally, adverse childhood experiences (Childhood Trauma Screener; CTS) are assessed at t1 and treatment satisfaction (ZUF-8) at t2.

Ecological momentary assessments (EMAs) will be conducted weekly using a newly developed questionnaire consisting of a set of five items. EMAs are used to track participants’ current condition (quality of life, physical/psychological well-being, concentration, everyday abilities).

As an additional part of the study, blood samples are taken at t1 and t2 to analyse changes in the IL-1Beta, IL6, TNF cytokine triad which has been associated with Long/Post-COVID-sequelae in a recent study (29).

Blood sampling is performed only for those participants who have additionally given their consent for this part of the study.

### Participant timeline

**Figure 1:**
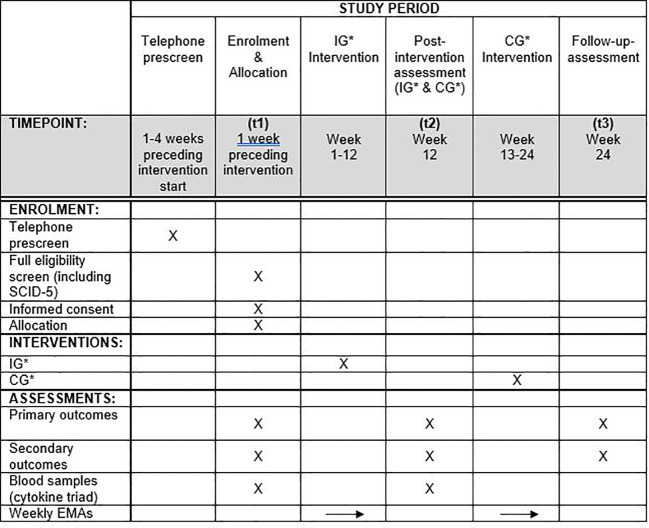
Schematic schedule for participants. *IG, Intervention group; CG, Control group.

A schematic schedule for participants is depicted in **Figure 1**.

### Sample size

A total of N = 120 participants will be assigned to the study arms, 60 to the waitlist with regular care arm and 60 to the PsyLoCo intervention arm. Case numbers are based on feasibility within a pilot trial.

### Recruitment

Patients will be recruited via specialised Long/Post-COVID outpatient clinics of the participating university hospitals, via general practitioners or via the programme’s website (https://psyloco.de/).

## Assignment of interventions: allocation

### Sequence generation

The allocation sequence will be generated in *R* using the *blockrand* package (https://cran.r-project.org/web/packages/blockrand/index.html). The randomization is stratified by study center. The allocation sequence is created using blocking with varying block sizes (4 and 6).

### Concealment mechanism

The allocation sequence will be stored in a CSV-file in a folder that is only accessible by the biometrician who creates the allocation sequence. It is uploaded to the randomization tool in RedCap. Only the responsible biometrician has access to the file. Once the project starts, the allocation sequence cannot be changed or accessed by anyone.

### Implementation

The allocation sequence will be generated by a biometrician at the Institute for Medical Epidemiology, Biometry and Informatics at the Martin-Luther-University Halle Wittenberg. The enrolment and assigning of participants will be done by the study personnel at the five study sites.

## Data collection and management

### Plans for assessment and collection of outcomes

The variables of interest are assessed via digital self-assessment questionnaires in LimeSurvey. The only exception are the cytokine values which will be examined through blood sample analysis. Blood samples are taken by certified medical personnel.

### Plans to promote participant retention and complete follow-up

Participants can terminate study participation at any time. In this case no further outcome data are collected. To ensure completion of follow-up, participants will be contacted via e-mail and reminded to fill out the questionnaire at one week and two weeks after the initial request to complete the follow-up-questionnaire. If, after three weeks, the questionnaire has not yet been completed, participants will be given a final reminder via a telephone call.

### Data management

Since data collection is digital via LimeSurvey and RedCap, the data is entered directly by the participants/therapists and exported from the databases as CSV-file without person-identifying data for use in the statistical program *R*. Both RedCap and LimeSurvey are hosted on secure servers of the Central Service “Information and Communication” (ZB IuK) of the University Clinic Halle. Only pseudonymised data is exported for analysis.

### Confidentiality

Participant data will be collected pseudonymised. For data analysis, data will be exported to the statistical program *R*. Only the study administrator can access the list containing pseudonymisation information and digitally gathered participant health-related data.

### Plans for collection, laboratory evaluation and storage of biological specimens for genetic or molecular analysis in this trial/future use

Plasma will be separated from whole blood by centrifugation at 1400 x g for 10 min. Remaining cell debris will be removed by high-speed centrifugation at 12000 x g for 10 min. Plasma samples will be stored at -80°C. Plasma cytokine levels will be quantified using bead-based multiplex flow cytometry kits from Biolegend (LEGENDplex Human B cell Panel (13-plex), Human Anti-Virus Response Panel (13-plex)). Measurements will be performed on a BD FACSCelesta flow cytometer (compare (29) for more methodological details).

## Statistical methods

### Statistical methods for primary and secondary outcomes

The statistical analysis will be conducted utilizing descriptive analysis of primary and secondary outcomes (e.g. differences in mean and median). In an exploratory fashion, mixed-effect linear regression analysis adjusted for study site (random effect) will be used to check for differences between intervention and control groups regarding intraindividual differences in the primary outcome Health-Related Quality of Life (measured by SF-12) between enrolment (t1) and post-intervention assessment (t2). Covariates will be adjusted for if misbalance in characteristics at baseline assessment is more than 20%. In the same way, secondary outcomes will be analysed. These analyses will be also conducted to assess stability of changes i.e. comparing outcomes at t2 and t3. EMAs will be analysed using analysis of variance respectively trajectories approach to detect changes over time. Immunological parameters (i.e. cytokine levels) will be analysed in the same way as primary outcome – in case of non-normal distributions, transformation will be applied.

Additional analyses will be conducted regarding feasibility of recruitment, drop-out rate, therapist adherence and participant adherence.

### Methods for additional analyses (e.g subgroup analyses)

In exploratory fashion, subgroup analyses comparing males and females and younger and older participants, dichotomized at median, will be conducted for the planning of subgroup analysis in the later RCT.

### Methods in analysis to handle protocol non-adherence and any statistical methods to handle missing data

Both, intention-to-treat and per-protocol analysis will be conducted to assess the extent of protocol violation. Single items missing within the outcome instruments, respectively, will be imputed by median, if less than 20% of items are missing.

Therapies are planned to be held over the course of 12 weeks. In case of any cancelled appointments therapies can be prolonged for up to two weeks. Participants who drop out before session four of the intervention will be replaced. Four missed sessions will be treated as non-adherence.

### Plans to give access to the full protocol, participant level-data and statistical code

Given the pilot study approach, there is no plan to give access to the full protocol, participant level-data and statistical code.

## Oversight and monitoring

### Composition of the coordinating centre and trial steering committee

The study is coordinated at the institutions in Munich (TU), Magdeburg and Halle (Saale). Statistical and biomedical analyses are conducted at the Institute for Medical Epidemiology, Biometrics and Informatics at the university of Halle (Saale). Psychotherapeutic interventions are conducted at the psychosomatic departments of the university hospitals in Munich (TU), Magdeburg, Tuebingen, Ulm and Freiburg. At each study site an equal number of participants will be included (n=24). The coordinating staff meets regularly every two weeks. Full staff meetings are held monthly. Therapeutic sessions will be supervised in small groups of up to five therapists once a month by an experienced and senior specialist.

### Adverse event reporting and harms

Any adverse events will be documented. They will be discussed within the PsyLoCo consortium and reported to the ethical committees.

### Frequency and plans for auditing trial conduct

Annual project reports will be sent to the funding agency as requested.

### Plans for communicating important protocol amendments to relevant parties (e.g. trial participants, ethical committees)

Important protocol amendments will always be communicated to ethical committees of all participating locations of the PsyLoCo consortium.

## Dissemination plans

The pilot trial’s results will be published in peer reviewed journals. Additionally, the results will be disseminated to health care professionals at national and international conferences as well as to the public in appropriately suitable media of the lay press.

## Discussion

The PsyLoCo pilot trial is designed to gain insights into the feasibility and estimate the efficacy of a specialized modular psychotherapeutic treatment for Long/Post-COVID patients. The various applied measures are implemented to determine if participants benefit from the intervention overall and/or in certain areas of their lives. Long/Post-COVID can be an intense and persistent condition associated with a variety of limitations in life and consequently a noticeable psychosocial burden (10). Complementary to an often unidimensional, somatic view on Long/Post-COVID and a resulting one-dimensional treatment, multimodal treatment approaches including treatment for psychological and psychosocial needs should be applied. This is especially true as psychotherapeutic approaches have proven helpful in treating medically unexplained bodily symptoms (22, 23).

In addition to measuring psychometric data, the PsyLoCo-study also aims to record biological parameters (cytokine triad IL-1 Beta, IL6, TNF) and their changes during the intervention. Cytokine trials could indicate phenotypes with differential response to interventions.

### Strengths and limitations

Strengths of the study are a thorough psychometric screening as well as the conduct of a structured clinical interview to confirm potential psychological diagnoses. Second, the wide range of instruments used, demonstrates the variety of complaints of those affected in a very comprehensive way. Third, the distribution of treatment centers throughout the country evens out potential regional differences regarding psychosocial needs. This increases the generalizability of the results. Another strength is the integrative psychotherapeutic manual which was developed in an iterative process by an interdisciplinary team of all participating sites. With the 12-session approach, the manual conceptualizes a possibility of a time-efficient treatment option.

Limitations that have to be mentioned and that are connected to the nature of pilot study designs are firstly the relatively small sample size with 120 participants. Secondly, different COVID-19 variants have been shown to lead to a different severity of Long/Post-COVID symptomatology (30). Examining whether the therapeutic treatment is particularly effective for a certain virus variant subgroup of patients is beyond the scope of the study, but should be subject of future studies. Another limitation is the lack of neurocognitive testing to objectify potential neurocognitive complaints of patients.

### Trial status

Protocol version 1.0. Recruitment began mid of March 2023 and ended mid of June 2023. The successful submission of the protocol was delayed by the incorrect description of pilot status in the previous version.

Trial registration: The trial has been registered with the German Clinical Trials Register (Deutsches Register Klinischer Studien; Trial-ID: DRKS00030866) on March 7, 2023.

## Ethics statement

The studies involving humans were approved by Ethikkommission Technische Universität München. The studies were conducted in accordance with the local legislation and institutional requirements. The participants provided their written informed consent to participate in this study.

## Author contributions

CA: Conceptualization, Funding acquisition, Supervision, Writing – original draft, Writing – review & editing, Resources. TF: Conceptualization, Project administration, Writing – original draft, Writing – review & editing. PBr: Writing – original draft, Writing – review & editing. AD: Writing – review & editing, Methodology, Supervision. MB: Writing – review & editing. HW: Writing – review & editing. ME: Writing – review & editing. KG: Writing – review & editing, Conceptualization. MS: Writing – review & editing. HG: Writing – review & editing, Conceptualization. LW: Writing – review & editing. JK: Writing – review & editing. CL: Writing – review & editing, Conceptualization. AM: Writing – review & editing. PBe: Writing – review & editing. JM: Formal analysis, Writing – review & editing, Methodology. RM: Conceptualization, Data curation, Formal analysis, Funding acquisition, Methodology, Software, Writing – original draft, Writing – review & editing. FJ: Conceptualization, Funding acquisition, Writing – original draft, Writing – review & editing, Methodology, Supervision.
